# Fatty Acids and a High-Fat Diet Induce Epithelial–Mesenchymal Transition by Activating TGFβ and β-Catenin in Liver Cells

**DOI:** 10.3390/ijms22031272

**Published:** 2021-01-28

**Authors:** Oliwia Kwapisz, Judyta Górka, Agata Korlatowicz, Jerzy Kotlinowski, Agnieszka Waligórska, Paulina Marona, Natalia Pydyn, Jurek W. Dobrucki, Jolanta Jura, Katarzyna Miekus

**Affiliations:** 1 Department of General Biochemistry, Faculty of Biochemistry, Biophysics and Biotechnology, Jagiellonian University, 30-387 Krakow, Poland; oliwia.glabica@doctoral.uj.edu.pl (O.K.); judyta.gorka@doctoral.uj.edu.pl (J.G.); agata.korlatowicz@wp.pl (A.K.); j.kotlinowski@uj.edu.pl (J.K.); paulina.marona@doctoral.uj.edu.pl (P.M.); natalia.pydyn@doctoral.uj.edu.pl (N.P.); jolanta.jura@uj.edu.pl (J.J.); 2 Department of Cell Biophysics, Faculty of Biochemistry, Biophysics and Biotechnology, Jagiellonian University, 30-387 Krakow, Poland; agnieszka.waligorska@uj.edu.pl (A.W.); jerzy.dobrucki@uj.edu.pl (J.W.D.)

**Keywords:** high-fat diet, EMT, TGFβ, β-catenin, liver, MCPIP1

## Abstract

Nonalcoholic fatty liver disease is defined as the accumulation of excessive fat in the liver in the absence of excessive alcohol consumption or any secondary cause. Although the disease generally remains asymptomatic, chronic liver inflammation leads to fibrosis, liver cirrhosis, and even to the development of hepatocellular carcinoma (HCC). Fibrosis results from epithelial–mesenchymal transition (EMT), which leads to dedifferentiation of epithelial cells into cells with a mesenchymal-like phenotype. During EMT, epithelial cells with high expression of E-cadherin, influenced by growth factors, cytokines, and inflammatory processes, undergo morphological changes via enhanced expression of, e.g., vimentin, fibronectin, and N-cadherin. An inducer of EMT and, consequently, of fibrosis development is transforming growth factor beta (TGFβ), a pleiotropic cytokine associated with the progression of hepatocarcinogenesis. However, the understanding of the molecular events that direct the development of steatosis into steatohepatitis and liver fibrosis remains incomplete. Our study revealed that both prolonged exposure of hepatocarcinoma cells to fatty acids in vitro and high-fat diet in mice (20 weeks) result in inflammation. Prolonged treatment with fatty acids increased the levels of TGFβ, MMP9, and β-catenin, important EMT inducers. Moreover, the livers of mice fed a high-fat diet exhibited features of liver fibrosis with increased TGFβ and IL-1 levels. Increased expression of IL-1 correlated with a decrease in monocyte chemoattractant protein-induced protein 1 (MCPIP1), a negative regulator of the inflammatory response that regulates the stability of proinflammatory transcripts encoding IL-1. Our study showed that a high-fat diet induced EMT by increasing the levels of EMT-activating transcription factors, including Zeb1, Zeb2, and Snail and changed the protein profile to a profile characteristic of the mesenchymal phenotype.

## 1. Introduction

Epithelial–mesenchymal transition (EMT) is a process that drives the dedifferentiation of epithelial cells into cells with a mesenchymal-like phenotype. During EMT, intercellular connections are lost; the motility and invasive potential of cells increase due to activation of EMT inducers such as the Slug, Snail, Zeb 1/2, or Twist transcription factors; and E-cadherin expression is lost and replaced by expression of the mesenchymal cell marker N-cadherin [1]. An important inducer of the EMT process and the pro-invasive gene expression profile is β-catenin, which binds to T cell and lymphoid enhancer (TCF–LEF) factors [2,3]. In epithelial liver cells, EMT can generate mesenchymal/fibroblastic cells, which could be relevant in the progression of liver fibrotic diseases [4].

Fibrosis is a common outcome of chronic liver diseases (CLDs) characterized by tissue remodeling, an inflammatory environment, and altered molecular signaling pathways and is a result of long-term exposure to damaging agents [4]. Nonalcoholic fatty liver disease (NAFLD) is currently considered the most common chronic liver disease in developed countries. NAFLD encompasses a spectrum of well-defined stages from simple nonalcoholic fatty liver (NAFL) through nonalcoholic steatohepatitis (NASH) to cirrhosis in the absence of alcohol abuse. The understanding of the molecular events controlling the development and progression of NAFLD that direct the development of steatosis into steatohepatitis and liver fibrosis is still incomplete. Hepatic accumulation of lipids leading to lipotoxicity, activation of inflammatory cascades, and fibrogenesis, as well as multiple other insults acting together may lead to the development and progression of NAFLD [5,6,7,8].

Fibrosis can also result from a reparative or reactive process that can occur in the lung, kidney, heart, or liver and is characterized by the presence of excess fibrous connective tissue in an organ. Under chronic pathological conditions, fibrosis progresses to advanced states that lead to defective organ function and finally to organ failure [9,10]. An inducer of fibrosis development is transforming growth factor beta (TGFβ), whose expression is increased markedly in mesangial cells before kidney fibrosis, in fibroblasts from patients with pulmonary fibrosis, and in the cirrhotic liver [11,12]. TGFβ expression is also associated with morphologic alterations such as EMT in hepatocytes [13]. Increased levels of TGFβ1 have been linked to EMT in biliary epithelial cells before the progression of liver disease to hepatic fibrosis [11]. In addition, EMT itself plays a relevant role in the appearance of a profibrotic fibroblast phenotype during liver fibrosis [4].

In this study, we investigated the influence of a high-fat diet (HFD) and free fatty acids (FFAs) on the expression of TGFβ and β-catenin and the regulation of EMT in the liver. Our study revealed that prolonged exposure of hepatocarcinoma cells to fatty acids and HFD in mice (20 weeks) increased the levels of TGFβ, β-catenin, and IL-1, important inducers of EMT. HFD also increased the levels of EMT-activating transcription factors, including Zeb1, Zeb2, and Snail and changed the protein profile to a profile characteristic of the mesenchymal phenotype. Our results show the multifaceted effect of fat on cells, leading to EMT and, consequently, the development of fibrosis in the liver.

## 2. Results

### 2.1. Free Fatty Acids (FFA) Treatment Induces TGFβ Expression and Activates MMP9

**Figure 1:**
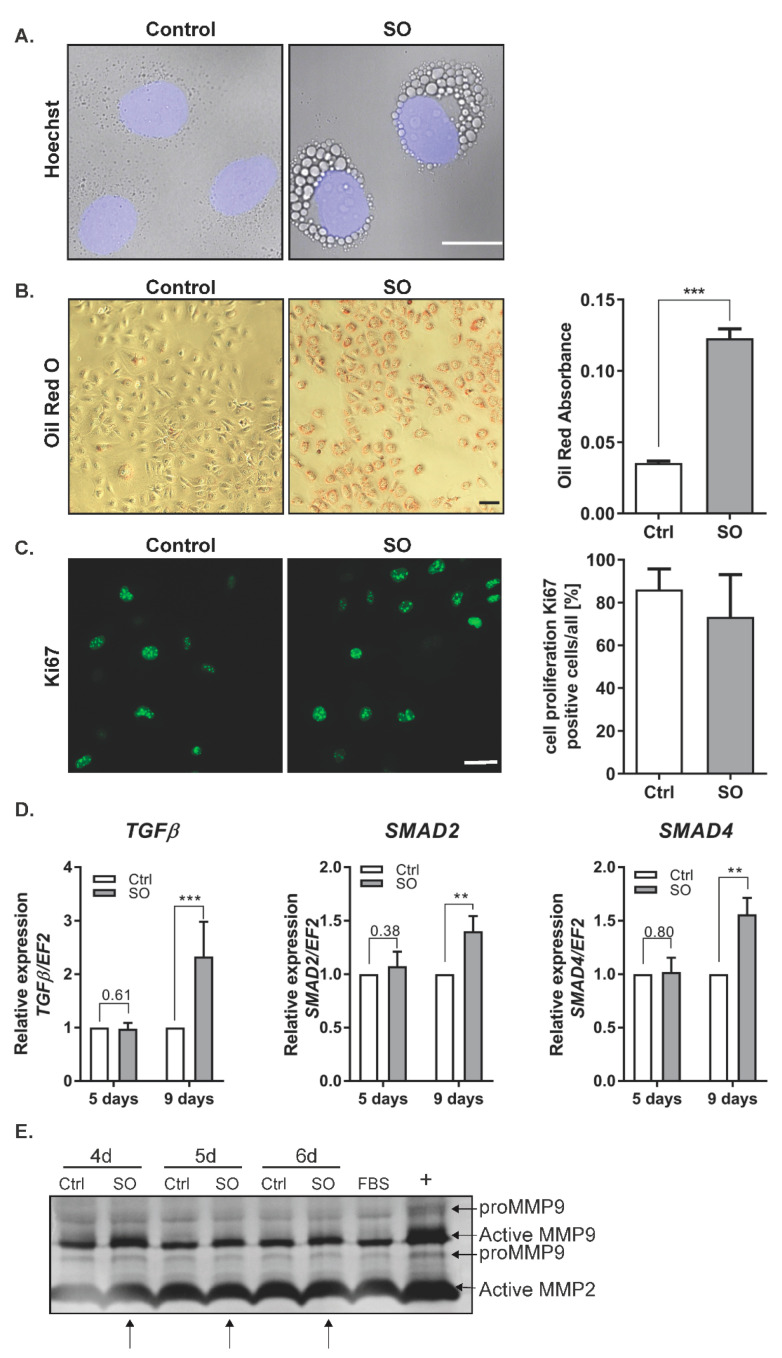
The influence of sodium oleate on the Huh7 cells phenotype. (**A**) Changes in morphology (63× magnification, Hoechst for nuclei, scale bar: 20 µm), SO—0.5 mM sodium oleate. (**B**) Oil Red O staining of the Huh7 cells stimulated for 24 h with 0.5 mM sodium oleate (SO) (representative pictures, 10× magnification, scale bar: 50 µm), The graph shows the differences in fat accumulation by control and stimulated cells. (**C**) Immunofluorescence staining of the proliferation marker Ki67 (representative images, magnification 63×, scale bar: 50 µm). The chart represents the ratio of positive cells compared to whole cells from five fields of view, *n* = 100, SO—0.5 mM sodium oleate. (**D**) mRNA expression of *TGFβ, SMAD2, SMAD4* after five and nine days of sodium oleate stimulation. Analysis of *TGFβ* was conducted in six independent experiments, analysis of *SMAD2* and *SMAD4* in three independent experiments; mRNA level in each sample was analyzed in duplicates. The mRNA level in the control was set to 1. The *EF2* gene was used as reference. (**E**) MMP2 and MMP9 levels after four, five, and six days of sodium oleate stimulation. The image of the gel shows the pro-form and active form of MMPs. The medium from the Caki-1 cell-stimulated phorbol myristate acetate (PMA) was used as the positive control (+), arrows indicate an increase of activity; Ctrl—control, SO—0.5 mM sodium oleate. The results are presented as the means ± SD, the Student’s *t*-test was used to determine the *p*-value, ** *p* < 0.01, *** *p* < 0.001.

Increased free fatty acid (FFA) levels are linked with NAFLD [14]. Analysis of lipid accumulation in hepatocytic cell lines has shown the highest extent of steatosis in the cells treated with a monounsaturated fatty acid (FA), oleic acid, in comparison to palmitic acid [15]. In the first step, we evaluated the ability of Huh7 hepatocellular carcinoma (HCC) cells to accumulate lipids. The cells were incubated in the presence of 0.5 mM sodium oleate (SO) as described previously [16]. After 24 h, we observed clearly marked droplets of fat, located mainly in the vicinity of the cell nucleus (Figure 1A). Oil Red O staining confirmed fat accumulation by the cells (Figure 1B). The presence of fatty acids in the cell culture did not significantly influence cell proliferation, as confirmed by Ki67 staining (Figure 1C). The saturated FFA palmitate (PA) was shown to change the behavior of cancer cells, increasing their invasive ability and activating Wnt and TGFβ signaling [17]. In our study, we examined whether a monounsaturated FFA, SO, changes the transcript level of TGFβ in Huh7 cells. We observed that 9 days of incubation with SO significantly increased the expression of *TGFβ* and transcriptional factors activated by TGFβ signaling, *SMAD2* and *SMAD4* (Figure 1D). TGFβ treatment upregulates matrix metalloproteinase (MMP) 2 and MMP9 [18]. We observed increased activity of MMP9 in the zymography assay, with no differences in MMP2 activity after SO treatment (Figure 1E).

### 2.2. FFA Treatment Increases the Levels of β-Catenin

**Figure 2:**
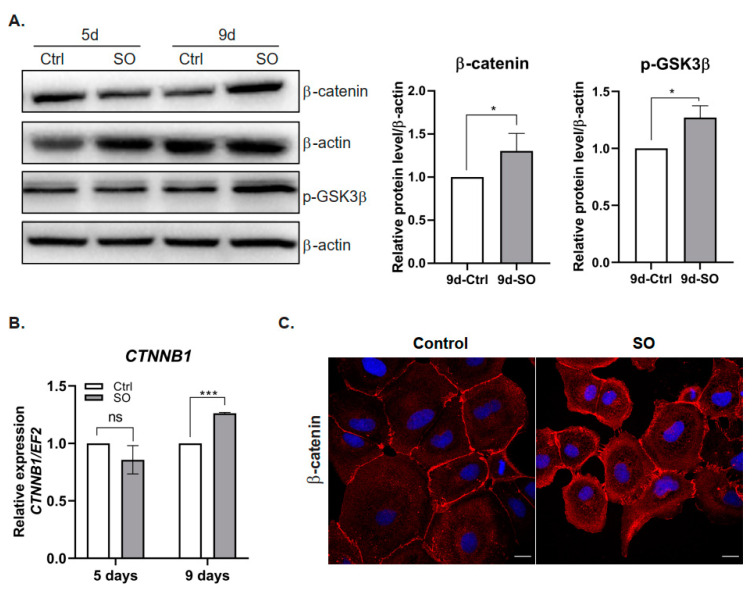
Effect of sodium oleate stimulation on the level of β-catenin in Huh7 cells. (**A**) Left panel, Western blot analysis of β-catenin and phosphorylated GSK3β protein levels in the cells stimulated with sodium oleate for five and nine days; β-actin was used as the loading control. Right panel, densitometric analysis from five (β-catenin) and three (GSK3β) independent repetitions: Huh7 cells after nine days of stimulation with sodium oleate, Ctrl—control, SO—0.5 mM sodium oleate. (**B**) mRNA expression of β-catenin (*CTNNB1*) after five and nine days of stimulation with sodium oleate, Ctrl—control, SO—0.5 mM sodium oleate. Analysis was conducted in three independent experiments and mRNA level in each sample was analyzed in duplicates. The mRNA level in the control was set to 1. The *EF2* gene was used as reference. (**C**) Representative images from a confocal microscope analysis of β-catenin (red) in the Huh7 cells stimulated with sodium oleate for five days. Primary antibody: mouse anti-β-catenin 1:50 (BD Biosciences), secondary antibody: Alexa Fluor 546 goat anti-mouse (Thermo Fisher Scientific, Waltham, MA, USA); Hoechst (blue) for nuclei, 63× magnification, scale bar, 20 µm. The results are presented as the means ± SD, the Student’s *t*-test was used to determine the *p*-value, * *p* < 0.05, *** *p* < 0.001, ns—non-significant.

TGFβ was shown to stimulate the canonical Wnt signaling pathway and nuclear accumulation of β-catenin, which resulted in the induction of fibrotic disease [19]. In our experimental model, the Huh7 cells treated with SO exhibited significantly increased protein and transcript levels of β-catenin (Figure 2A,B). Confocal microscopy analysis confirmed the accumulation of β-catenin in the cytoplasm and near the cell membrane after SO treatment (Figure 2C). Further analysis of β-catenin localization did not show nuclear accumulation of this protein as shown for HepG2 and Hep3B [17]. This protein was located in the cytoplasm and in the perinuclear region rather than in the nucleus (Figure 2C). We also found an increased level of the inactive (phosphorylated at serine 9 (Ser9)) form of glycogen synthase kinase (GSK) 3β after SO treatment (Figure 2A). Inhibition of GSK3β through phosphorylation at serine 9 led to the stabilization of β-catenin [20] observed in the Huh7 cells treated with SO for nine days (Figure 2A,C).

### 2.3. FFA Treatment Changes EMT Marker Expression in HCC Cells

**Figure 3:**
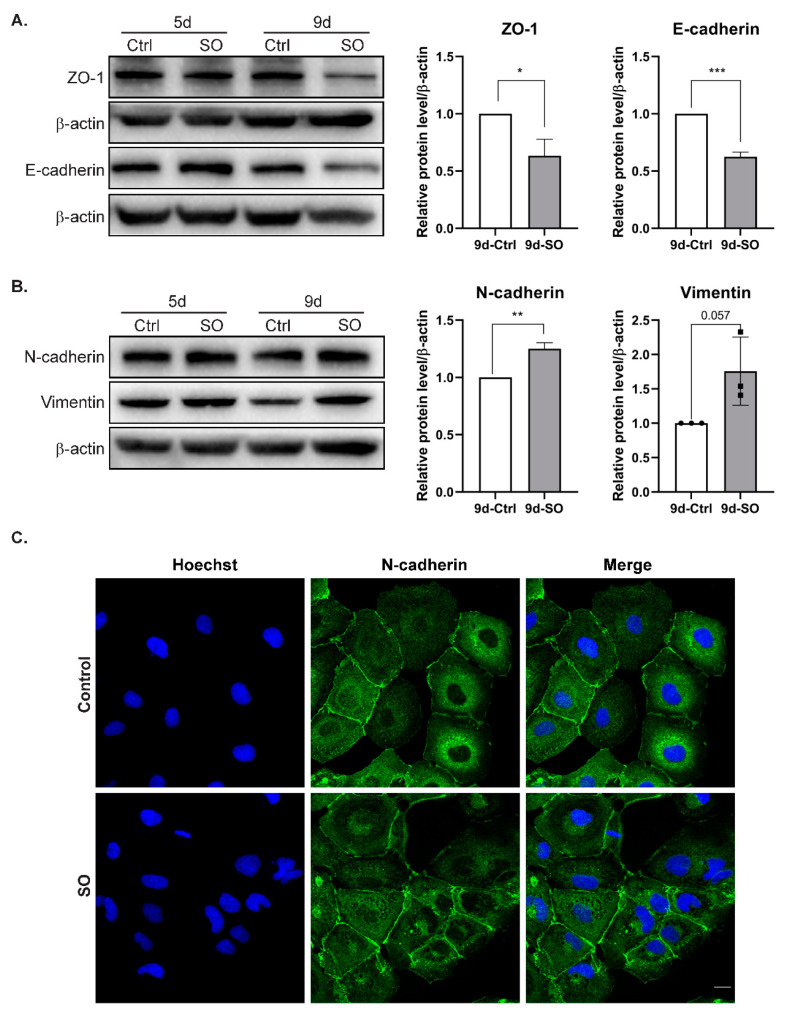
The effect of sodium oleate on the level of epithelial and mesenchymal markers in Huh7 cells after five and nine days of stimulation. (**A**) Analysis of epithelial markers (E-cadherin, ZO-1) level using the Western blot method. On the right, densitometric analysis from three independent repetitions: Huh7 cells after nine days of stimulation with sodium oleate, the control was set to 1, Ctrl—control, SO—0.5 mM sodium oleate. β-actin was used as the loading control. (**B**) Analysis of mesenchymal marker (N-cadherin, vimentin) levels using the Western blot method. On the right, densitometric analysis from three independent repetitions: Huh7 cells after nine days of stimulation with sodium oleate; the control was set to 1, Ctrl—control, SO—0.5 mM sodium oleate. β-actin was used as the loading control. (**C**) Representative images from a confocal microscope analysis of N-cadherin in the Huh7 cells stimulated with sodium oleate for five days (63× magnification, Hoechst for nuclei, scale bar, 20 µm). Primary antibody: rabbit polyclonal to N-cadherin 1:200 (Abcam), secondary antibody: Alexa Fluor 488 goat anti-rabbit (Thermo Fisher). The results are presented as the means ± SD, the Student’s *t*-test was used to determine the *p*-value, * *p* < 0.05; ** *p* < 0.01; *** *p* < 0.001.

It has already been shown that treatment with the saturated FFA palmitate increases the migration rate of the human liver cancer cell lines HepG2 and Hep3B. Moreover, elevated palmitate levels change expression levels of various EMT markers in liver cancer cells [17]. In our experimental model, we observed that treatment with SO decreased the levels of the epithelial markers E-cadherin and ZO-1 in Huh7 cells (Figure 3A). The protein levels decreased with incubation time and were the lowest after nine days of incubation with SO (Figure 3A). Triplicate densitometric analysis also showed that the decreases in the levels of epithelial cell markers were statistically significant (Figure 3A). The levels of structural proteins characteristic of the mesenchymal phenotype, such as N-cadherin and vimentin, increased after nine days of incubation with SO (Figure 3B). Immunofluorescence analysis after five days of incubation with SO showed no change in the levels of N-cadherin after 5-day SO treatment (Figure 3C).

### 2.4. High-Fat Diet or Oleic Acid Treatment Increases the Levels of TGFβ and IL-1β and Decreases MCPIP1

**Figure 4:**
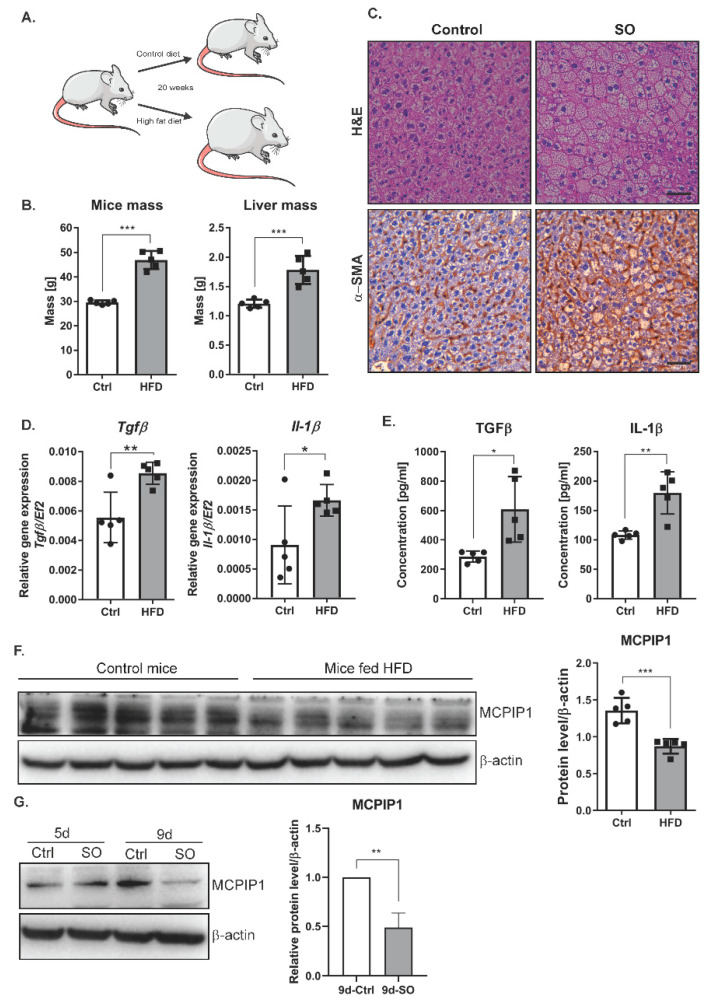
Phenotype of liver cells in the mice fed a high-fat diet (Ctrl, *n* = 5, HFD, *n* = 5). (**A**) The scheme of the experiment: 10-weeks-old C57BL/6J mice were fed a control or high-fat diet for 20 weeks. (**B**) Graphs showing mouse body and liver weight due to excessive fat intake. (**C**) Hematoxylin–eosin and α-SMA staining shows changes in the morphology of liver cells in the mice fed a high-fat diet. Immunohistochemical evaluation was performed using primary monoclonal anti-smooth muscle actin antibody (1:100, Dako, Glostrup, Denmark) and EnVision Detection Systems Peroxidase/DAB ((3,3′-Diaminobenzidine) Rabbit/Mouse (Dako), scale bar, 50 µm (**D**) mRNA expression estimated by real-time PCR for *Il-1β* and *TGFβ* in mouse livers, Ctrl—mice on a control diet, HFD—mice fed a high-fat diet. The dots represent individual animals, *n* = 5. The *EF2* gene was used as reference. (**E**) The protein levels of IL-1β and TGFβ in mouse livers were obtained using ELISA. Ctrl—mice on a control diet, HFD—mice fed a high-fat diet. The results are presented as the means ± SD. The dots represent individual animals, *n* = 5. (**F**) Left panel, representative Western blot of the MCPIP1 level in mouse livers. Ctrl—mice on a control diet, HFD—mice fed a high-fat diet. Right panel, densitometric analysis of the MCPIP1 level in mouse livers. The dots represent individual animals, *n* = 5. (**G**) Left panel, representative Western blot of the MCPIP1 level in the Huh7 cells stimulated with sodium oleate for five (5d) and nine (9d) days; β-actin was used as the loading control. Right panel, densitometric analysis of three independent experiments. The results are presented as the means ± SD, the Student’s *t*-test was used to determine the *p*-value, * *p* < 0.05; ** *p* < 0.01; *** *p* < 0.001.

Because prolonged treatment of Huh7 cells with SO increased the expression of TGFβ and EMT markers, we transferred these studies to an in vivo model. We investigated the livers of mice subjected to 20 weeks of an HFD. As expected, feeding mice an HFD (60% kcal from fat) for 20 weeks resulted in increased weight gain and liver mass compared with those in the animals fed a control diet (Figure 4A,B). Subsequently, we evaluated liver fibrosis in the mice fed an HFD or a control diet for 20 weeks. The deposition of α-smooth muscle actin (α-SMA), a marker for a subset of activated fibrogenic cells and tissue fibrogenesis [21], was clearly increased in the mice fed an HFD (Figure 4C, Supplementary Figure S1). HFD induced significant hepatic steatosis as assessed by hematoxylin and eosin staining (Figure 4C) as well as inflammation indicated by significantly increased hepatic expression of IL-1β and TGFβ at both the transcript (Figure 4D) and protein (Figure 4E) levels. An important regulator of IL-1β expression is monocyte chemoattractant protein-induced protein 1 (MCPIP1) [22]. In our experimental model, the MCPIP1 level in Huh7 cells decreased after prolonged treatment with SO (Figure 4F). Interestingly, the fibrotic livers observed in the mice fed an HFD were characterized by a significantly reduced level of MCPIP1 (Figure 4G). This decrease in MCPIP1 expression may also explain the observed increase in the level of IL-1 β (Figure 4D,E).

### 2.5. NAFLD in Mice Increases the Levels of β-Catenin and Activates the EMT Program

**Figure 5:**
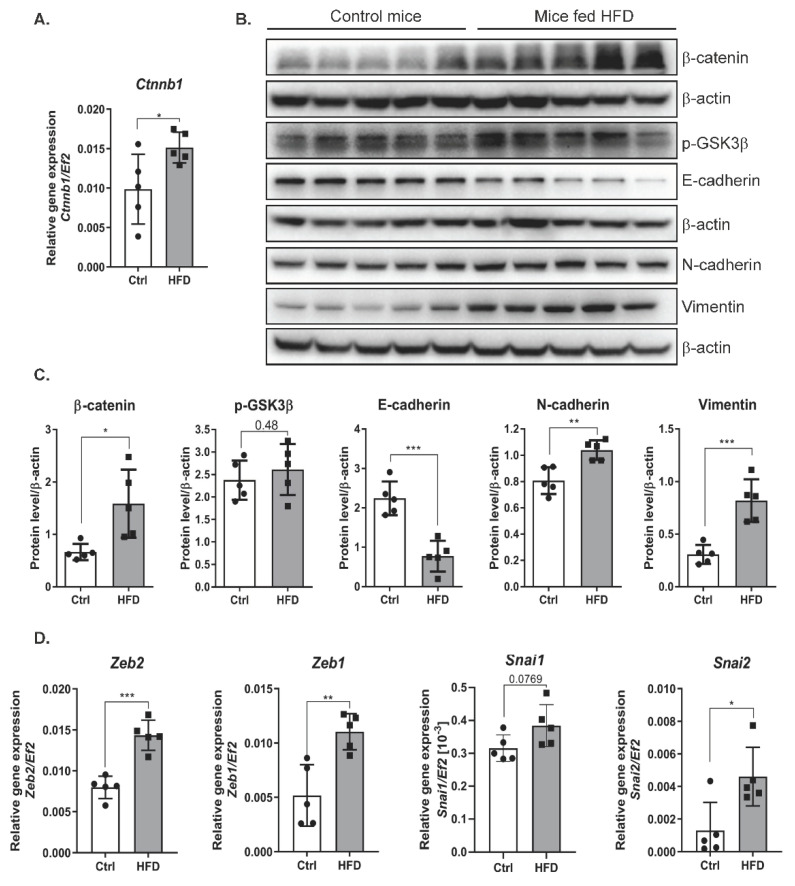
The effect of a high-fat diet on the level of epithelial and mesenchymal markers in liver cells of mice. (**A**) mRNA expression of β-catenin (*ctnnb1*) in livers of the mice fed a control or a high-fat diet. Ctrl—mice on a control diet, HFD—mice fed a high-fat diet. The dots represent individual animals, *n* = 5 The *EF2* gene was used as reference. (**B**) Analysis of epithelial and mesenchymal marker levels using the Western blot method. β-actin was used as the loading control. (**C**) Densitometric analysis of protein levels in mouse livers. Ctrl—mice on a control diet, HFD—mice fed a high-fat diet. The dots represent individual animals, *n* = 5. (**D**) The level of gene expression of transcription factors in mouse livers. Ctrl—mice on a control diet, HFD—mice fed a high-fat diet. The dots represent individual animals, *n* = 5. The *EF2* gene was used as reference. The results are presented as the means ± SD, the Student’s *t*-test was used to determine the *p*-value, * *p* < 0.05; ** *p* < 0.01; *** *p* < 0.001.

Interaction between the canonical Wnt pathway and TGFβ plays a key role in the pathogenesis of fibrotic diseases [19]. Our results showed that livers isolated from the mice fed an HFD had increased mRNA expression of β-catenin compared with those from the mice fed a control diet (Figure 5A). Consistent with the gene expression results, the hepatic protein levels of β-catenin as determined by Western blot analysis were increased in the livers of mice fed an HFD (Figure 5B). Similar to the in vitro results (Figure 2A), the level of the phosphorylated form of GSK3β was increased in the livers of mice fed an HFD (Figure 5B,C). Since an HFD activates the expression of TGFβ, an inducer of EMT, we investigated whether increased levels of TGFβ and activation of the β-catenin pathway contribute to EMT in the liver. HFD decreased the level of E-cadherin and increased the levels of the mesenchymal markers N-cadherin and vimentin (Figure 5B,C). In addition, HFD induced a significant increase in the expression of EMT-activating transcription factors. We observed higher levels of *Slug, Snail, Zeb1*, and *Zeb2* transcripts in the livers of mice fed a high-fat diet (Figure 5D).

## 3. Discussion

Chronic inflammatory reactions induced by a variety of stimuli may induce fibrosis development characterized by the presence of excess fibrous connective tissue in an organ. Progressive fibrosis resulting in organ destruction is a potential effect of EMT when injury and inflammation persist and results in fibroblastic cell accumulation [9]. EMT describes the process by which cells lose typical epithelial characteristics and acquire mesenchymal traits. EMT occurs when tissues are constructed during embryogenesis/development, during remodeling or fibrosis of adult tissues, and during tumor development. Currently, EMT is not considered a single isolated process but a set of multiple and dynamic transitional states between the epithelial and mesenchymal phenotypes. Interestingly, intermediate states have been identified as crucial drivers of organ fibrosis and tumor progression [23]. The results presented in this paper indicate that excessive fatty acid (i.e., FFA) influx leads to inflammation and activates EMT in vitro and in vivo via a mechanism mediated by increased levels of TGFβ, β-catenin and decreased levels of MCPIP1.

TGFβ is a pleiotropic cytokine that inhibits proliferation, suppresses transformation, and induces apoptosis during hepatocarcinogenesis [24]. On the other hand, TGFβ secretion has been associated with the progression of hepatocarcinogenesis [25]. TGFβ is also a major inducer of EMT during embryogenesis, cancer progression, and fibrosis [26,27,28]. We found that FFA increased the level of TGFβ in cultured hepatocarcinoma cells as well as in the livers of mice fed an HFD. Our results are consistent with previous research showing that HepG2 and Hep3B cells treated with the saturated FFA palmitate have increased expression of TGFβ [17]. Moreover, increased expression of TGFβ was found in patients during the progression of NASH-associated fibrosis [29]. TGFβ signaling has been shown to promote lipid accumulation in hepatocytes, inflammatory cell infiltration, hepatocyte death, and fibrosis [30]. In addition, our results showed that FFA treatment induced the activity of MMP9 in Huh7 cells. This finding is consistent with previous findings showing that TGFβ treatment upregulates MMP9 [18] and that on the other hand, MMP9 proteolytically cleaves latent TGFβ, thus providing a novel and potentially important mechanism for TGFβ activation [31], a master positive regulator of EMT [32].

**Figure 6:**
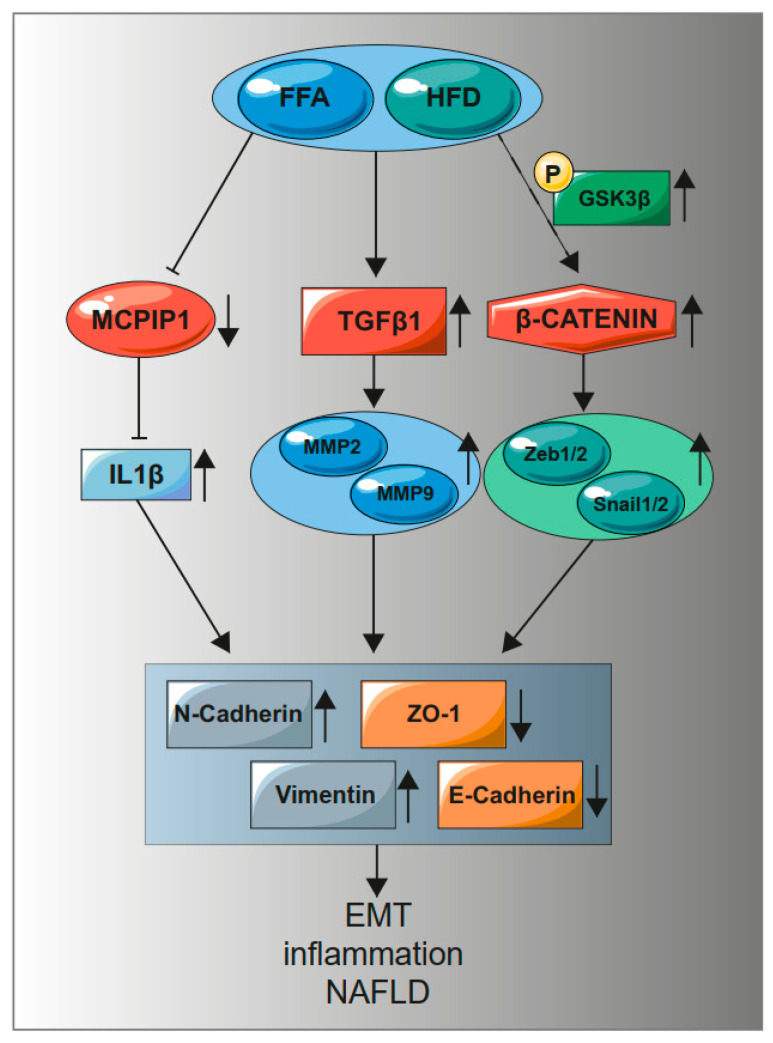
A high-fat diet and treatment with fatty acids increase the level of TGFβ1, β-catenin while reducing the level of MCPIP1. An increase in TGFβ1 causes an increase in MMP9. A decrease in MCPIP1 induces an increase in IL-1β, which increases mesenchymal markers with a simultaneous decrease in epithelial markers. Phosphorylation of GSK3β (S9) inactivates the β-catenin degradation complex, leading to activation of the genes dependent on the WNT/β-catenin pathway. Activation of MMP9, transcription factors Zeb1/2 and Snail1/2, an increase in IL-1β induce the EMT process, inflammation, and NAFLD.

We indicate that activation of the EMT process by FFA does not depend on one signaling pathway. We show that EMT causes changes in cells leading to the acquisition of a mesenchymal phenotype and may lead to fibrotic changes in the liver. The complexity of the observed processes is demonstrated in a diagram showing proposed mechanism of action of a high-fat diet and exposure of liver cancer cells to fatty acids (Figure 6).

Our studies revealed reduced levels of the epithelial markers E-cadherin and ZO-1 with simultaneously increased levels of N-cadherin and vimentin. N-cadherin is an indicator of ongoing EMT, and downregulation of E-cadherin is balanced by increased expression of mesenchymal N-cadherin, resulting in a “cadherin switch” [11]. In addition, palmitate treatment has been demonstrated to significantly reduce desmoplakin expression [33], which can lead to loss of cell–cell adhesion via desmosomes [34]. The decreased level of E-cadherin after prolonged exposure to FFAs in our experimental models may result from the elevated expression of Snail and ZEB1/2, the transcription factors that repress E-cadherin expression [35,36]. Additionally, it may result from increased levels of TGFβ, since this cytokine has been shown to be an activator of Snail expression [37], and Snail causes TGFβ-mediated repression of E-cadherin expression [38]. In hepatocytes and HCC cells, TGFβ upregulates Snail, which not only mediates EMT, but also suppresses TGFβ-induced apoptosis [39].

Our study demonstrated that FFA treatment or an HFD increased the level of β-catenin and altered its localization. A previous study showed that the canonical Wnt/β-catenin pathway is activated in fibrotic diseases and that TGFβ signaling activates the Wnt pathway [19]. Previous findings have also shown that TGFβ and β-catenin/cAMP-response element-binding protein-binding protein (CBP)-dependent pathways regulate α-SMA induction [40]. Our study demonstrated that prolonged exposure to FFAs in vitro and in vivo significantly increased β-catenin expression at both the mRNA and protein levels. This finding is consistent with previous findings showing that the overall expression levels of β-catenin were increased in both HepG2 and Hep3B cells after palmitate treatment [17]. However, in contrast, our confocal microscopy analysis did not show increased nuclear localization of β-catenin. In contrast, this protein was located near the cell membrane, in the cytoplasm, and in the perinuclear region rather than in the nucleus. β-Catenin has been shown to be overexpressed in hepatic fibrosis [41]. Increased levels of β-catenin may result from activation of TGFβ, because TGFβ signaling decreases the expression of Dkk-1 and activates the Wnt pathway [19]. Moreover, the canonical Wnt/β-catenin signaling pathway inactivates GSK3β, preventing β-catenin phosphorylation and inactivation [42]. In our study, we observed that FFA treatment in vitro and in vivo increased the levels of phosphorylated GSK3β, which cannot inactivate β-catenin.

β-Catenin has been shown to bind directly to the ZEB1 promoter and activate its transcription in colorectal carcinomas, and the β-catenin/TCF4 complex induces the EMT activator ZEB1 to regulate tumor invasiveness [43]. In our study, increased β-catenin levels correlated with increased expression of ZEB1 in the livers of mice fed an HFD. Our results show the multifaceted effect of fat on cells, leading to EMT and, consequently, the development of fibrosis in the liver.

The understanding of the molecular events that direct the development of steatosis into steatohepatitis and liver fibrosis remains incomplete. However, inflammation mediated through cytokine signaling pathways, including IL-1 signaling, might be the link between steatosis and steatohepatitis [44]. IL-1β promotes hepatic steatosis by stimulating triglyceride and cholesterol accumulation in primary liver hepatocytes and inducing lipid droplet formation [45]. Deficiency of either IL-1α or IL-1β is sufficient to protect against NASH development [44]. However, an HFD increases hepatic expression of IL-1β [30]. IL-1β has also been shown to enhance TGFβ1-driven EMT in vitro, highlighting another potential mode of action for this cytokine [46]. Considering these data, the level of TGFβ in fatty livers or cells treated with FFA may be indirectly regulated by MCPIP1. MCPIP1 degrades IL-1 transcripts, and when the level of this protein is low, IL-1 expression increases. Moreover, we have already shown that MCPIP1 regulates the levels of MMP9 and TIMP3 [47], and a similar mechanism may be active in liver tissue.

In our model, we found a significant increase in the transcript and protein levels of IL-1 in the livers of mice fed an HFD. Moreover, our study revealed decreased levels of the MCPIP1, which directly regulates IL-1 expression [22]. A previous study showed that MCPIP1 also contributes to lipid metabolism in hepatocytes. Prolonged treatment of HepG2 cells with SO was shown to decrease the level of MCPIP1 [16]. Moreover, diet-induced obesity leads to liver steatosis accompanied by a decreased amount of the MCPIP1 in murine hepatocytes [16]. On the other hand, our previous study showed that a low level of MCPIP1 is associated with acquisition of the mesenchymal phenotype in clear cell renal cell carcinoma cells [48].

Cell line-based experiments, however, have limitations. Human primary hepatocytes are the gold standard for investigating lipid metabolism in nonalcoholic fatty liver disease (NAFLD). However, they present issues with availability, inter-donor variability, and the short time frame during which they remain differentiated [49,50]. As a result, proliferating human hepatoma cell models, the hepatoma cell lines Huh7 and HepG2, are the most widely used options [51].

These results indicate a multifaceted effect of fat on the development of fibrosis in the liver (Figure 6). Excessive accumulation of lipids in liver cells activates inflammation, as manifested by an increase in the level of IL-1 and a decrease in the level of its regulator MCPIP1. The inflammatory process leads to an increased level of TGFβ and activation of β-catenin signaling pathways promoting EMT, which leads to acquisition of mesenchymal features and induces hepatic fibrosis.

## 4. Materials and Methods

### 4.1. Cell Culture and Stimulation with Free Fatty Acids

A human hepatoma cell line (Huh7) was generously contributed to our research by Mr. Michelangelo Foti of the Department of Cell Physiology and Metabolism, University of Geneva. The cells were cultured at 37 °C in a 5% CO_2_ atmosphere using Dulbecco’s Modified Eagle’s Medium-low glucose (1 g/L) (DMEM-LG; Lonza, Walkersville, MD USA) supplemented with 2 mM L-glutamine (Lonza) and 10% fetal bovine serum (FBS, Sigma-Aldrich, St. Louis, MO, USA). The cells were seeded in 6/12-well culture plates in confluence 10% and 30%. On the following day, the cells were stimulated with 0.5 mM sodium oleate (SO, Sigma-Aldrich) for five and nine days with a medium change every 48 h. Sodium oleate was dissolved in 10 mM NaOH to the concentration of 80 mM. Separately, a mix of the DMEM with 2 mM fatty acid-free BSA (bovine serum albumin) was prepared in the 9:10 ratio. Both solutions were heated to 70 °C and 50 °C, respectively. Afterwards, they were mixed together and incubated for 15 min at 50 °C. The medium with NaOH was used as a control. A detailed description of the stimulation with sodium oleate has been previously described by Pydyn et al. [16].

### 4.2. Oil Red O Staining

The cells were fixed by overnight incubation in 4% formaldehyde, washed with 60% isopropanol, and incubated for 10 min in 2.1% Oil Red (Sigma-Aldrich) solution. Images were taken with Nicon Eclipse in the bright field at a magnification of 10×. The dye was finally extracted with 100% isopropanol to measure the absorbance of the samples at 500 nm using a Tecan Spectra Fluor Plus Microplate Reader (Tecan, Männedorf, Switzerland).

### 4.3. Ki-67 Immunofluorescent Staining

The cells were plated on a glass coverslip in 6-well culture plates. On the following day, the cells were stimulated with 0.5 mM sodium oleate with a change of medium every 48 h. After nine days, the cells were fixed in 4% paraformaldehyde. Subsequently, the cells were permeabilized by 1% Triton X-100 in phosphate-buffered saline (PBS) and blocked with 0.2% Triton X-100 in 1% BSA in PBS followed by an overnight incubation with the Ki-67 antibody (Abcam, Cambridge, UK) diluted 1:100. Afterwards, the samples were incubated for 1 h with the goat anti-rabbit Alexa Fluor 488 secondary antibody (Thermo Fisher Scientific, Waltham, MA, USA) and mounted with the ProLong Gold Antifade reagent with DAPI ((4′,6-diamidino-2-phenylindole, Thermo Fisher Scientific). Images of the cells were acquired using the Leica LAS X (Leica Application Suite X) image acquisition software and a Leica DMC5400 fluorescence microscope (Leica Microsystem, Wetzlar, Germany) with a 63× immersion objective.

### 4.4. Animal Studies

The experiments were conducted in accordance with the guidelines of the II Local Ethics Committee of the Institute of Pharmacology of the Polish Academy of Sciences (approval No. 82/2019, date of approval 18/4/2019). The mice were handled according to the regulations of the national and local animal welfare bodies under SPF (Specific-pathogen-free) conditions, with sufficient water and food provided at all times. The experiment was conducted on 10-week-old C57BL/6J mice who were fed a normal or a high-fat diet (containing 60% kcal from fat, Zoolab, Sedziszow, Poland) for a period of 20 weeks. The material collected from the livers was immersed in formalin or stored in −80 °C. Caudate lobe, left and right lateral lobes were stained with hematoxylin–eosin to show the liver morphology.

### 4.5. RNA Isolation and Quantitative RT-PCR

RNA from cells was isolated using a Gene MATRIX Universal RNA Purification Kit (EURx, Gdansk, Poland) according to the manufacturer’s instructions for cell culture. RNA from livers was isolated from the left median lobe using the Fenozol–chloroform method (A&A Biotechnology, Gdynia, Poland). The concentration of RNA was measured using NanoDrop 2000 (Thermo Fisher Scientific). Reverse transcription was performed using 1 µg of RNA, M-MLV (Moloney Murine Leukemia Virus) reverse transcriptase, and buffer 10× (Promega, Madison, WI, USA), dNTP (deoxyribonucleotide triphosphate, EURx), oligo(dT) (Sigma-Aldrich, Haverhill, UK). The reaction was carried out for 5 min at 65 °C, 1 h at 42 °C, and 15 min at 72 °C. Real-time PCR was performed using QuantStudio3 (Thermo Fisher Scientific), RT PCR Mix SYBR A, and 10 µM primers (Sigma-Aldrich). The transcript level was calculated by the ΔΔCT method and elongation factor 2 (EF2) was used as a reference. The primer sequences and annealing temperatures are shown in the Supplementary Table S1. Analysis was conducted in three independent experiments and mRNA level in each sample was analyzed in duplicates.

### 4.6. Western Blot Analysis

The protein lysate from cells was isolated using the Mammalian Protein Extraction Reagent (MPER; Thermo Fisher Scientific) with protease and phosphatase inhibitors for 10 min and then centrifuged for 20 min, 4 °C, 14,000× *g*. The protein lysate obtained from livers (left median lobe) was isolated using a RIPA (Radioimmunoprecipitation assay buffer) solution with protease and phosphatase inhibitors. After 30 min of incubation on ice, the lysates were centrifuged for 20 min, 4 °C, 10,000× *g*. The electrophoresis separation was carried out in 10% polyacrylamide gel. After the transfer, PVDF (Polyvinylidene fluoride) membranes (Merck-Millipore, Burlington, MA, USA) were blocked for 1 h in 3% BSA in Tris-buffered saline with 0.1% Tween (TBS-T) followed by an overnight incubation in the primary antibody at 4 °C. On the following day, the membranes were rinsed with TBS-T and incubated for 1 h with the secondary antibody. The Immobilon TM Western Chemiluminescent HRP Substrate (Merck-Millipore) and the ChemiDoc system (Bio-Rad, Hercules, CA, USA) were used for signal detection. The list of antibodies is shown in the Supplementary Table S2. Analysis was conducted in three independent experiments. In densitometric quantification, the control was set to 1, and the results are presented as the means ± SD of three independent experiments.

### 4.7. ELISA

The lysates for ELISA were isolated from the right median lobe in 1% Triton in PBS with protease inhibitors, then centrifuged for 10 min, 4 °C, 10,000× *g*. To evaluate levels of cytokines in mouse livers (TGFβ and IL-1β), DuoSet ELISA assays were performed (R&D Systems, Minneapolis, MN, USA) according to the manufacturer’s instructions. Absorbance was measured at 450 nm; 540 nm was used as reference using a Tecan Spectra Fluor Plus Microplate Reader (Tecan, Männedorf, Switzerland).

### 4.8. Confocal Analysis

Coverslip cultures were fixed in 4% paraformaldehyde, permeabilized by 1% Triton X-100 in PBS, and blocked with 0.2% Triton X-100 in 1% BSA in PBS. The primary antibody was incubated at 4 °C overnight, the secondary antibody—for 1 h in the dark at room temperature. Primary antibody N-cadherin dilution, 1:200 (Abcam, Cambridge, UK), β-catenin—1:100 (BD Biosciences, Franklin Lakes, NJ, USA), secondary antibody Alexa Fluor—1:1000 (Thermo Fisher Scientific). Images of the cells were acquired using a Leica SP5 confocal system with the sequential mode of scanning. An oil immersion 63× objective numerical aperture 1.4 was used. Hoechst 33342 fluorescence was induced with a 405 nm laser and collected by a PMT set in the range of 415–470 nm; Alexa Fluor 488-marked N-cadherin antibodies were excited with a 488 nm line of an argon laser, collected in the range of 500–540 nm, and Alexa Fluor 546 with β-catenin antibodies were excited with a 543 nm HeNe laser, collected in the range of 565–700 nm.

### 4.9. Immunohistochemical Staining

Tumor sections were stained with hematoxylin–eosin to visualize tumor morphology. Immunohistochemical evaluation was performed using primary monoclonal anti-smooth muscle actin antibody (1:100, Dako) and EnVision Detection Systems Peroxidase/DAB, Rabbit/Mouse (Dako, Glostrup, Denmark). All images were taken using a Leica DMC5400 fluorescence microscope with a 10× objective with the Leica LAS X image acquisition software.

### 4.10. Gelatin Zymography

The cells were plated on a glass coverslip in 6-well culture plates. On the following day, the cells were stimulated with 0.5 mM sodium oleate with a change of medium every 48 h. After four, five, and six days, the medium from the cells was collected. The medium from the Caki-1 cell-stimulated phorbol myristate acetate (PMA) was used as the positive control. The gelatin zymography was performed using the protocol provided by Abcam (https://www.abcam.com/protocols/gelatin-zymography-protocol).

### 4.11. Statistical Analysis

The in vitro experiments were performed in three independent trials, all results are presented as the means ± SD. Unpaired Student’s *t*-test was used for comparison of the two groups, * *p* < 0.05; ** *p* < 0.01; *** *p* < 0.001.

## Data Availability

The data that support the findings presented in this manuscript are available from the corresponding author upon request.

## Supplementary Materials

The following are available online at https://www.mdpi.com/1422-0067/22/3/1272/s1, Figure S1: The effect of high-fat diet on the deposition of α-smooth muscle actin (α-SMA), Table S1: Primers sequences, Table S2: List of antibodies.
