# Supplementary material for: Fatty Acids and a High-Fat Diet Induce Epithelial–Mesenchymal Transition by Activating TGFβ and β-Catenin in Liver Cells
Source: Int J Mol Sci. 2021 Jan 28;22(3):1272. doi: 10.3390/ijms22031272 (PMC7865431; doi:10.3390/ijms22031272)
Supplement: Supplementary file 1 [file ijms-22-01272-s001.zip › ijms-1075374-SI/Supplementary Figure 1.pdf]

A.

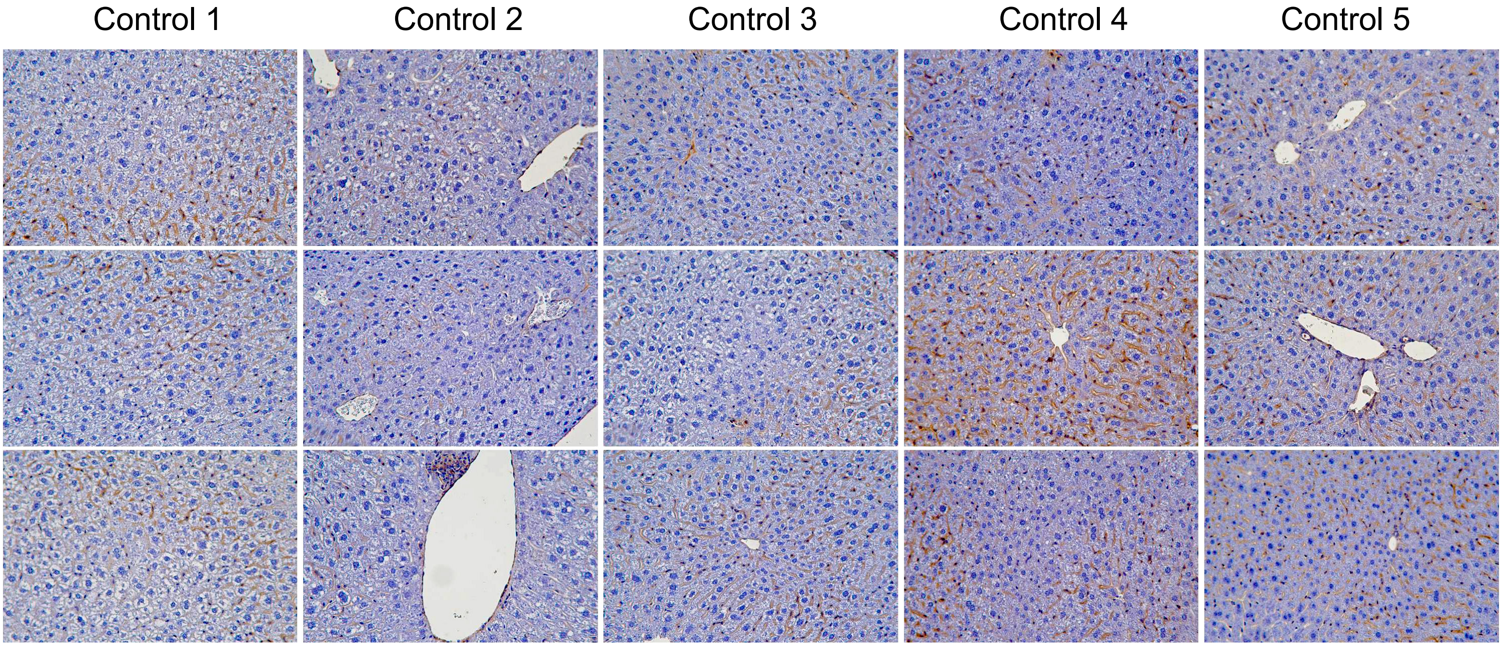

Control 1
Control 2
Control 3
Control 4
Control 5

B.

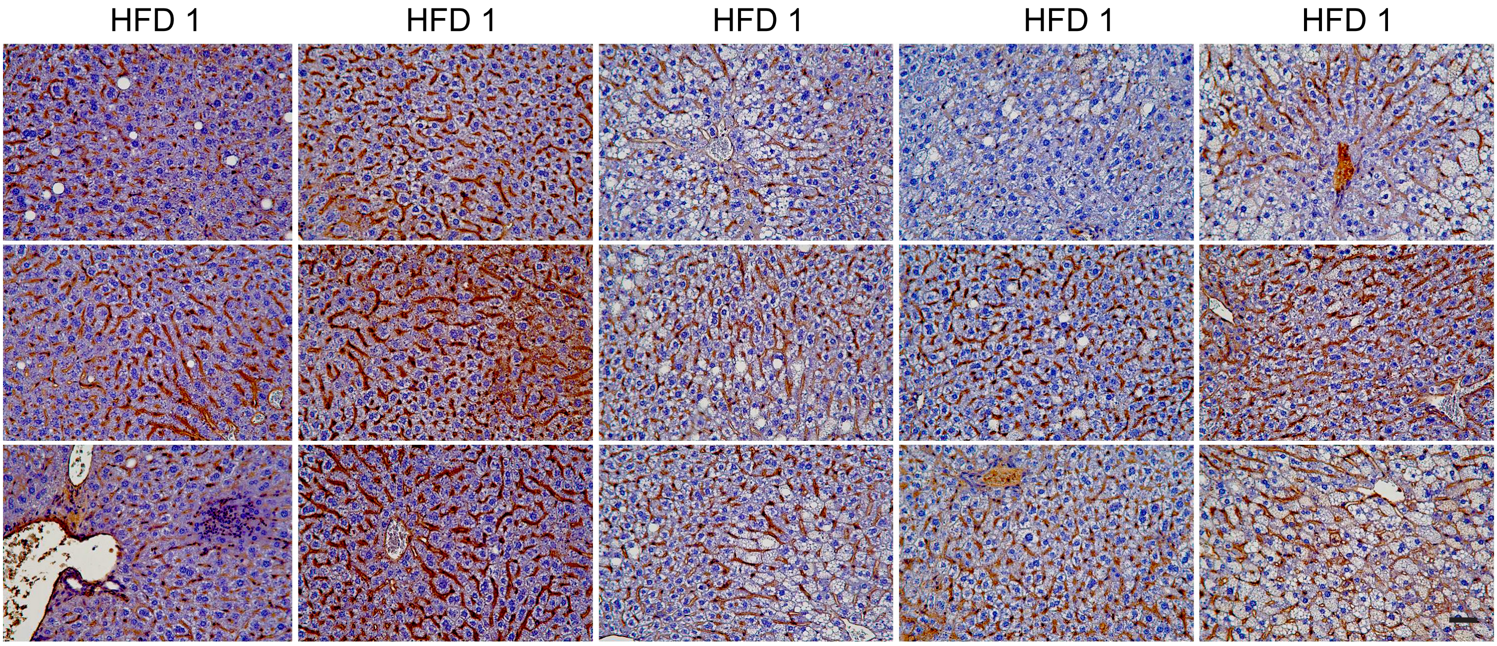

HFD 1
HFD 1
HFD 1
HFD 1
HFD 1

Supplementary Figure 1

The effect of high-fat diet on the deposition of α-smooth muscle actin (α-SMA).

Hematoxylin-eosin and α-SMA staining shows changes in the morphology of liver cells in mice fed a high-fat diet. Immunohistochemical evaluation was performed using primary monoclonal anti-smooth muscle actin antibody (1:100, Dako) and EnVision Detection Systems Peroxidase/DAB, Rabbit/Mouse (Dako). 5 mice on control diet, 5 mice fed high-fat diet (HFD), three fields of view, scale bar 50 μm.
