# Supplementary material for: Fatty Acids and a High-Fat Diet Induce Epithelial–Mesenchymal Transition by Activating TGFβ and β-Catenin in Liver Cells
Source: Int J Mol Sci. 2021 Jan 28;22(3):1272. doi: 10.3390/ijms22031272 (PMC7865431; doi:10.3390/ijms22031272)
Supplement: Supplementary file 1 [file ijms-22-01272-s001.zip › ijms-1075374-SI/Supplementary Table 1 and 2.docx]

Table 1 Primers sequences.

| Gene | Sequence |
| --- | --- |
| *TGFB* | F 5’ GGA CAT CAA CGG GTT CAC TAC 3’ |
|  | R 5’ TGA GAA GCA GGA AAG GCC G 3’ |
| *CTNNB1* | F 5’ AAA ATG GCA GTG CGT TTA G 3’ |
|  | R 5’ TTT GAA GGC AGT CTG TCG TA 3’ |
| *SMAD2* | F 5’ AGT GTG TAA AAT TCC ACC AG 3’ |
|  | R 5’ ATT CTA GTT AGC TGA TAG ACG G 3’ |
| SMAD4 | F 5’ AAA GGT CTT TGA TTT GCG TC 3’ |
|  | R 5’ CTA TTC CAC CTA CTG ATC CTG 3’ |
| *Il1b* | F 5’ GGA TGA TGA TGA TAA CCT GC 3’ |
|  | R 5’ CAT GGA GAA TAT CAC TTG TTG G 3’ |
| *Tgfb1* | F 5’ GGA TAC CAA CTA TTG CTT CAG 3’ |
|  | R 5’ TGT CCA GGC TCC AAA TAT AG 3’ |
| *Ctnnb1* | F 5’ GAT TAA CTA TCA GGA TGA CGC 3’ |
|  | R 5’ TTA TTA ACT ACC ACC TGG TCC 3’ |
| *Snai2* | F 5’ GAC ACA TTA GAA CTC ACA CTG 3’ |
|  | R 5’ GAC ATT CTG GAG AAG GTT TTG 3’ |
| *Snai1* | F 5’ AGT TGA CTA CCG ACC TTG 3’ |
|  | R 5’ AAG GTG AAC TCC ACA CAC 3’ |
| *Zeb1* | F 5’ATA TGA GCA CAC AGG TAA GAG 3’ |
|  | R 5’ TTC ATG TGT TGA GAG TAG GAG 3’ |
| *Zeb2* | F 5’GAG ACT TTA CTC CAG AAT TGG 3’ |
|  | R 5’ ATG AGC CTC AGG TAA TAA AGG 3’ |

Table 2 List of antibodies.

| Antibody | Producer | Dilution |
| --- | --- | --- |
| Rabbit anti-phospho-GSK-3β (Ser9) | Cell Signaling | 1:1000 |
| Mouse anti-β-catenin | BD Biosciences | 1:1000 |
| Mouse anti-β-actin | Sigma | 1:4000 |
| Rabbit anti-ZO1 | GeneTex | 1:1000 |
| Mouse anti-E-cadherin | BD Biosciences | 1:500 |
| Rabbit anti-N-cadherin | Abcam | 1:1000 |
| Rabbit anti-vimentin | Abcam | 1:1000 |
| Rabbit anti-MCPIP1 | GeneTex | 1:2000 |
